# Plasma exchange plus glucocorticoids in the treatment of immune checkpoint inhibitor‐induced myocarditis: A case series and review

**DOI:** 10.1002/clc.24149

**Published:** 2023-09-12

**Authors:** Guibao Ke, Peian Chen, Jing Luo, Junlin Huang, Yuqi Shang, Yongzhang Huang, Ningying Fu, Hongbo Peng, Yuan Li, Bo Wang, Weijie Guan, Yonghua Peng, Xiaomin Yu, Jie Xiao

**Affiliations:** ^1^ Department of Nephrology The First Affiliated Hospital of Guangzhou Medical University Guangzhou China; ^2^ Department of Nephrology Affiliated Hospital/Clinical Medical College of Chengdu University Chengdu China; ^3^ Department of Critical Care Medicine Maoming People's Hospital Maoming China; ^4^ Department of Nephrology West China Hospital of Sichuan University Chengdu China; ^5^ State Key Laboratory of Respiratory Disease, Department of Thoracic Surgery and Oncology The First Affiliated Hospital of Guangzhou Medical University, National Clinical Research Center for Respiratory Disease Guangzhou China

**Keywords:** glucocorticoids, immune checkpoint inhibitors, myocarditis, plasma exchange

## Abstract

Immune checkpoint inhibitors (ICIs), including antiprogrammed cell‐death (PD)‐1/anti‐PD‐ligand (PDL‐1) monoclonal antibodies, are effective at improving the prognosis of patients with cancer. Among immune‐related adverse events, myocarditis associated with anti‐PD‐1/anti‐PD‐L1 antibodies is rare but lacks effective treatment and mortality is very high. In this study, the authors extracted data from the previous 8 years from electronic medical records housed in the hospital information system to identify patients hospitalized with myocarditis putatively caused by anti‐PD‐1/anti‐PD‐L1 tumor therapy. Clinical data from these patients are reported. Four patients who developed myocarditis after undergoing treatment with anti‐PD‐1/anti‐PD‐L1 antibodies for malignant tumors, all of whom responded favorably to therapy consisting of plasma exchange and glucocorticoids for myocarditis, and all patients improved and were discharged from hospital. Plasma exchange plus systemic glucocorticoids may be effective for treating anti‐PD‐1/anti‐PD‐L1 antibody‐induced myocarditis in patients with cancer.

## Graphical abstract

Among immune‐related adverse events, myocarditis associated with anti‐PD‐1/anti‐PD‐L1 antibodies is rare but lacks effective treatment and mortality is very high. In this study, the authors successfully cured four patients using plasma exchange plus systemic glucocorticoids. This case series suggests a novel approach to the treatment of immune checkpoint inhibitor‐induced myocarditis.

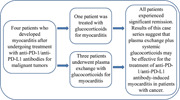

## 1 BACKGROUND

Immune checkpoint inhibitors (ICIs), which fight cancer cells by inducing T cell activation, have revolutionized cancer treatment over the past decade and have been used to treat nearly 50% of cancer types.^1^ ICIs, which include anti‐programmed cell death‐1 (PD‐1) and anti‐PD‐1 ligand (PD‐L1) antibodies, are widely used in the treatment of solid and hematological malignancies to improve overall survival and serve as an important treatment option for advanced cancers.^2^, ^3^ However, they can also induce immune‐related adverse effects (iRAEs) in a wide variety of tissues, causing myocarditis, pneumonitis, and/or other related conditions.^4^, ^5^ In particular, ICI‐induced myocarditis, although rare, can result in mortality in nearly 50% of affected patients.^6^, ^7^

The present case series describes four patients who developed myocarditis after undergoing treatment with anti‐PD‐1/anti‐PD‐L1 antibodies for malignant tumors, all of whom responded favorably to therapy consisting of plasma exchange and glucocorticoids for myocarditis.

## 2 CASE PRESENTATIONS

**Figure 1:**
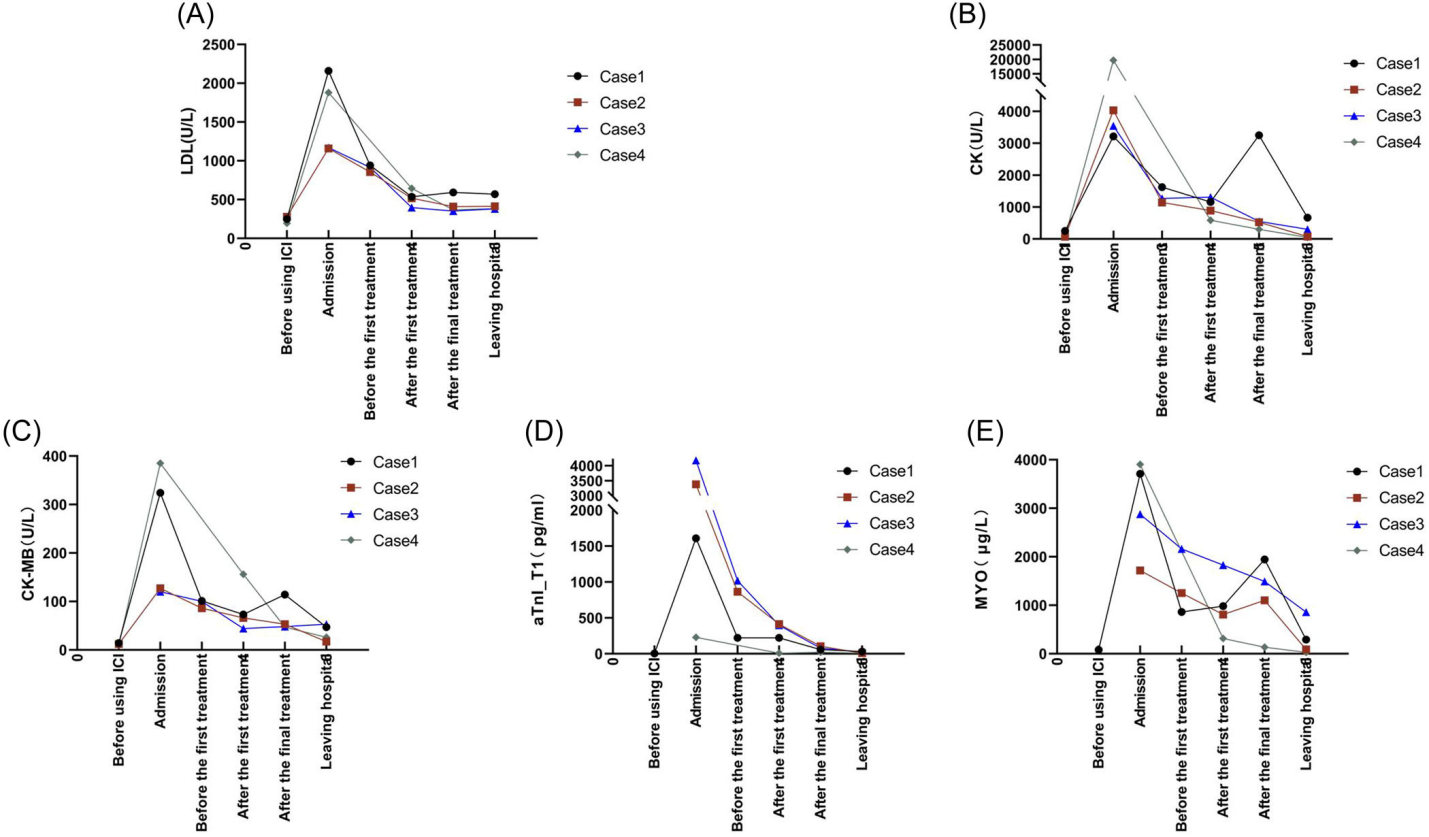
(A‐E) Changes in blood levels of lactate dehydrogenase (LDH), creatine kinase (CK), creatine kinase isoenzyme (CK‐MB), ultrasensitive troponin 1 (aTnI_T1) and myoglobin (MYO) at treatment time points in four patients. Treatment consisted of synthetic therapy based on plasma exchange in cases 1–3, and synthetic therapy based on glucocorticoids in case 4.

Four patients, ranging in age from 52 to 59 years, who developed myocarditis after treatment with anti‐PD‐1 and anti‐PD‐L1 antibodies are described. The tumor pathology, comorbidities, anti‐PD‐1/anti‐PD‐L1 antibody therapy, and plasma exchange combined with the glucocorticoid treatment regimens are summarized in Table 1. All patients were treated with glucocorticoids, and three underwent plasma exchange with glucocorticoids for myocarditis. All patients improved and were discharged from hospital. During hospitalization, laboratory indices suggestive of myocardial injury, including lactate dehydrogenase (LDH), creatine kinase (CK), creatine kinase isoenzyme (CK‐MB), ultrasensitive troponin 1 (aTnI_T1), and myoglobin (MYO) were examined. Changes in these indices in the four patients (cases 1–4) are shown in Figure 1. It is evident that these indices were drastically reduced after therapy.

**Table 1:** Clinical characteristics of four patients.

Patient	Gender	Age, years	Tumors	Tumor pathology	Comorbidity	ICI treatment plan	Diagnosis of myositis	Treatment modality	Number of plasma exchange
1	Female	59	Thymoma	Type B2 thymoma	Hypertension	Tislelizumab 200 mg, once every 3 weeks	Myocarditis and myositis associated with immune checkpoint inhibitor therapy	Plasma exchange + glucocorticoids + IVIg	5
2	Female	53	Thoracic cancer	B2/B3 mixed type thymic malignancy	None	Penpulimab 200 mg, once every 3 weeks	Myocarditis and myositis associated with immune checkpoint inhibitor therapy	Plasma exchange + glucocorticoids + IVIg	3
3	Male	55	Thymoma	B1/B3 mixed type thymoma	None	Tislelizumab 200 mg, once every 3 weeks	Myocarditis and myositis associated with immune checkpoint inhibitor therapy	Plasma exchange + glucocorticoids + IVIg + pyridostigmine + mycophenolate mofetil	3
4	Male	52	Lung cancer	Keratinizing squamous cell carcinoma	None	Sintilimab 200 mg, once every 3 weeks	Myocarditis and myositis associated with immune checkpoint inhibitor therapy	Glucocorticoids + IVIg	0

Abbreviation: IVIg, intravenous immunoglobulin.

### 2.1 Case 1

A 59‐year‐old woman presented with sudden weakness without an overt cause in June 2021, which progressively worsened with back pain, drooping of the left eyelid, and blurred vision in the left eye. She was diagnosed with thymoma in 2015 after a mediastinal tumor was identified during a routine physical examination. In August 2015, the patient underwent resection of a mediastinal tumor and a lung mass in the left upper lobe. After the relapse of the thymoma, she underwent surgical resection between September 2018 and April 2021. Pathological examination revealed type B2 thymoma with multiple pleural metastases and implants. She denied experiencing dark haze, photophobia, myalgia, a distorted mouth, chest tightness or pain, palpitations, dysphagia, or dyspnea.

The patient began receiving tislelizumab injections for thymoma in May 2021. Symptoms relapsed despite a course of tislelizumab. On admission, laboratory investigations revealed the following: LDH, 2158.6 U/L; CK, 3214.2 U/L; CK‐MB, 324.0 U/L; aTnI_T1, 1610.10 pg/mL; and MYO, 3712.6 g/L. There were no significant abnormalities in cardiac structures or blood flow, and measurements of left ventricular systolic function were normal. Electromyography (EMG) revealed active myogenic damage, damage to multiple peripheral nerves, and diaphragmatic myogenic damage. Unfortunately, no muscle biopsies were performed. Myocarditis and myositis associated with ICI therapy were strongly suspected.

She was treated with methylprednisolone 500 mg once daily and immunoglobulin 20 g/day for 5 days after hospital admission. She then underwent five courses of plasma exchange (2000 mL per session) while receiving methylprednisolone and immunoglobulin. The glucocorticosteroid dose was gradually decreased until maintenance therapy (20 mg/day) was achieved. The patient eventually recovered and was discharged from hospital. All laboratory findings indicated a trend toward recovery after treatment.

### 2.2 Case 2

A 53‐year‐old woman presented with shortness of breath with no overt cause in September 2021. She experienced nocturnal paroxysmal dyspnea, but no major signs of discomfort. In January 2016, she underwent resection of a mediastinal mass with pathological findings suggestive of B2/B3 mixed thymoma.

On August 30, 2021, the patient was administered penicillin. Her symptoms relapsed after 10 days of treatment. On admission, laboratory investigations revealed the following: LDH, 1159.2 U/L; CK, 4031.0 U/L; CK‐MB, 127.0 U/L; aTnI_T1, 3382.6 pg/mL; and MYO, 171 g/L. There were no significant structural or blood flow abnormalities within the heart, and left ventricular systolic function was normal. EMG revealed active myogenic damage in the left anterior tibialis, left and medial femoral head muscles, right biceps, and right deltoid muscles. Neurogenic myasthenia gravis was diagnosed based on muscle biopsy. Myocarditis and myositis associated with ICI therapy was strongly suspected.

She was administered a combination of methylprednisolone (1000 mg/day) and immunoglobulin (20 g/day) 5 days after admission. The glucocorticoid and immunoglobulin doses were tapered and maintained at 20 mg/day and 10 g/day, respectively. Her cardiac enzyme levels were better on recheck compared with admission. During hospitalization, the patient underwent three courses of plasma exchange for further treatment. The patient was eventually discharged with symptom amelioration and a trend toward normalization of laboratory parameters.

### 2.3 Case 3

A 55‐year‐old man presented with dizziness, palpitations, chest tightness, shortness of breath, nausea, dyspepsia, weakness in both lower limbs, and blurred vision in August 2021. The patient's symptoms worsened over time. He previously had a B1/B3 mixed thymoma and was diagnosed with thymoma relapse in March 2020. He underwent regular chemotherapy (unknown regimen) before initiating treatment with tislelizumab (dose unknown) in July 2021, after which he developed symptoms.

Electrocardiography revealed complete right bundle branch block and laboratory investigations revealed elevated levels of cardiac enzymes, acetylcholine receptor antibodies, and antiskeletal muscle receptor complex kinase antibodies. The patient was then transferred to another hospital where he was diagnosed with myositis associated with ICI therapy. After treatment with methylprednisolone (1 g) for 2 days and immunoglobulin (10 g) for 1 day, his symptoms improved; however, he still experienced difficulty with opening his right eye.

**Figure 2:**
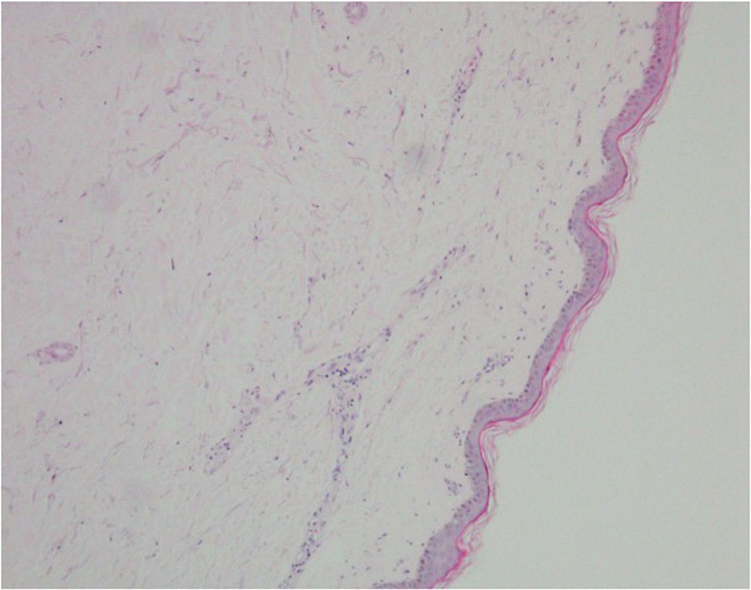
Pathology sample of muscle tissue biopsy from case 3, demonstrating partial cellular detachment and distortion, as well as perimysial lymphocytic infiltration.

After admission, laboratory investigations revealed the following: LDH, 1169.2 U/L; CK, 3544.1 U/L; CK‐MB, 120.0 U/L; aTnI_T1, 4180.30 pg/mL; and MYO, 2877.3 g/L. Pathological biopsies of the left anterior tibialis muscle and right biceps revealed lymphocyte infiltration around small vessels in the dermis, collagen fiber hyperplasia, cellular distraction and deformation, and sparse lymphocyte infiltration surrounding the muscle (Figure 2). Myocarditis and myositis associated with ICI therapy was suspected. He underwent three courses of plasma exchange (2000 mL per session) and 1 day of intravenous methylprednisolone (250 mg), followed by 40 mg daily maintenance. Simultaneously, he was administered 90 mg oral pyridostigmine three times daily and 0.75 g of mycophenolate mofetil twice daily. The patient was discharged from hospital.

### 2.4 Case 4

A 52‐year‐old man presented with cough and hemoptysis after a lung biopsy in July 2020, which revealed keratinizing squamous cell lung carcinoma. In October 2020, the patient was treated with sintilimab, paclitaxel, and platinum. In December 2020, he developed chest tightness, dyspnea, difficulty with raising his head, and bilateral ptosis. The patient was subsequently diagnosed with myocarditis and myositis associated with ICI therapy.

**Figure 3:**
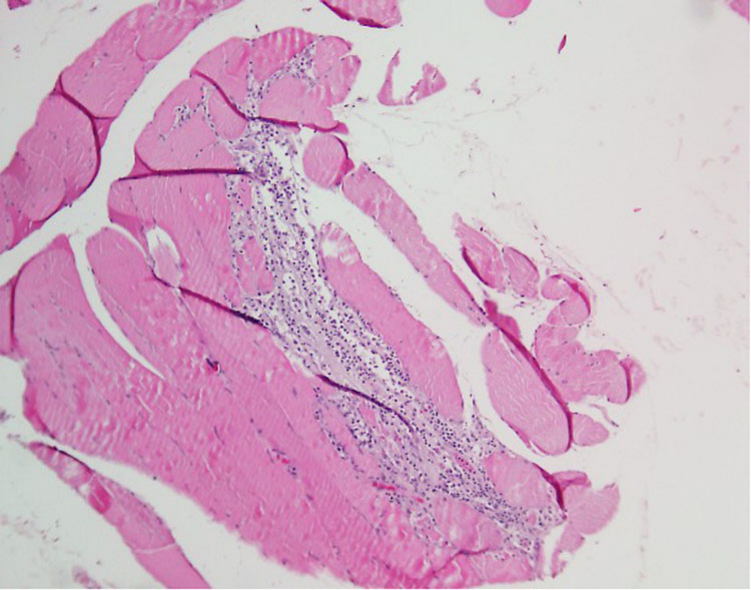
Pathology specimen of deltoid muscle biopsy from case 4, which exhibits focal muscle fiber atrophy with interstitial lymphocytic infiltration.

After admission, laboratory investigations revealed the following: LDH, 1880.6 U/L; CK, 19709.2 U/L; CK‐MB, 385.0 U/L; aTnI_T1, 227.5 pg/mL; and MYO, 3904.0 g/L. Skeletal muscle biopsy revealed focal muscle fiber atrophy with interstitial lymphocytic infiltration (Figure 3). After 2 days of high‐dose methylprednisolone treatment (240 mg/day), the dose was gradually reduced to 40 mg daily. The patient received immunoglobulin (10 g/day) for 5 days after admission. The patient was eventually discharged with symptom amelioration and a trend toward normalization of laboratory parameters.

## 3 DISCUSSION

Although myocarditis associated with anti‐PD‐1/anti‐PD‐L1 antibody treatment is rare, the mortality rate is very high and there are insufficient clinical data and established treatment strategies. Owing to the lack of specific signs and symptoms at onset, early detection is prone to under‐ and/or misdiagnosis.^8^, ^9^ In this study, we successfully cured four patients using plasma exchange plus systemic glucocorticoids.

### 3.1 Mechanism

ICIs, including anti‐PD‐1/anti‐PD‐L1 antibodies, are important therapeutic options for patients with cancer. According to the current theory, the recognition and clearance of tumor cells require the involvement of inflammatory cells from the innate and acquired immune systems, primarily CD8‐positive (+) T lymphocytes, CD4+ T lymphocytes, and macrophages. When immune cells are activated to fight cancer, antigens from cancer cells are recognized by antigen‐presenting cells (APCs).^10^ T‐cell receptors then bind to APCs via the cancer antigen major histocompatibility complex. However, CD28 (expressed on the T cell surface), which binds to B7‐1 and B7‐2 (CD80/CD86) of APCs, exerts the greatest influence on T cell responses. This is the second co‐stimulatory signal necessary to induce a T cell response. Co‐inhibitory molecules are key regulatory molecules that prevent T cell overactivation. PD‐1, PD‐L1, and cytotoxic T‐lymphocyte antigen‐ 4 (CTLA‐4) compete with positive costimulatory signals because they are important immune checkpoint pathways.^11^

PD‐1, a transmembrane protein with inhibitory properties that binds to PD‐L1 and PD‐L2, is one of these “immunodetecting” molecules. The binding of PD‐1 to PD‐L1/L2 inhibits tumor cell apoptosis, promotes the conversion of effector T cells into T regulatory cells (Tregs), and suppresses the activity of CD8+ T cells, reducing their ability to maintain tumor cell dormancy.^12^ Anti‐PD‐1/anti‐PD‐L1 antibodies are widely used in the treatment of solid and hematological malignancies, improving overall survival and serving as an important treatment option for advanced cancers. However, they can also induce iRAEs in a wide variety of tissues, causing myocarditis, and/or pneumonitis.^13^

According to data from the Bristol‐Myers Squibb corporate safety database, 18 drug‐related severe myocarditis adverse events (0.087%) occurred in 20 594 patients receiving nivolumab and/or ipilimumab.^14^ ICI‐induced myocarditis, although rare, can result in nearly 50% mortality in affected patients.^6^, ^7^ Here, we sought to characterize ICI‐induced myocarditis mechanistically. However, the pathophysiological mechanisms underlying ICI‐induced myocarditis are not completely understood. Nevertheless, a few studies have suggested that muscle‐specific antigens, including troponin and myosin, share characteristics between the tumor and cardiomyocytes,^15^ which could cause cross‐reactivity with T cells targeting both the tumor and cardiac muscle. Whole‐transcriptome sequencing has revealed high expression of inflammatory T cytokines and muscle‐specific transcripts in tumors, suggesting shared epitope(s) between tumors and striated muscles. Johnson et al.^14^ and Reuben et al.^16^ also reported that ICIs may cause myocarditis by co‐expressing specific antigens targeted by T cells in tumor cells, cardiomyocytes, and skeletal muscle cells. In addition, normal cells with PD‐L1 ligands expressed on their surfaces are protected from CD8+T cell (CTL)‐activated programmed cell death. Tumor cells have PD‐L1/L2 surface receptors, which keep them from undergoing programmed cell death, in support of the “immune escape” hypothesis. The autoimmune system upregulates PD‐L1 in skeletal and cardiac myocytes of patients with cancer, sparing CTL recognition and causing programmed cell death. In general, ICI myocarditis is associated with T cell and macrophage infiltration into the muscles and myocyte death.^9^

### 3.2 Management

Previous studies have consistently demonstrated that anti‐PD‐1 and anti‐PD‐L1 antibodies cause various adverse effects.^17^, ^18^, ^19^ The main cardiotoxicity associated with anti‐PD‐1/anti‐PD‐L1 antibodies is myocarditis.^20^, ^21^, ^22^, ^23^

Plasma exchange aims to rapidly remove antigens, antibodies, immune complexes, and cytokines from the plasma using a blood cell separation device, thus suppressing exaggerated cellular and humoral immunity and avoiding damage associated with over‐regulated immunity.^24^ Plasma exchange can supplement electrolytes, proteins, coagulation factors, and other blood components in patients. Therefore, it is used to treat numerous diseases characterized by a deficiency in protective components or the presence of harmful circulating factors. This is a novel approach for treating ICI‐induced myocarditis caused by ICIs. Plasma exchange can be used not only for the treatment of severe decline in cardiac function or cardiogenic shock caused by myocarditis^25^, ^26^ but also in combination with glucocorticoids for the treatment of myocarditis caused by ICIs,^1^ which may confer better efficacy than glucocorticoid therapy alone. However, due to the small number of cases, our conclusions cannot be generalized to other patient populations. As such, further studies are required to confirm these findings.

In this study, immunoglobulins were used in three patients. Immunoglobulins may contain specific antibodies against autoimmune antibodies in addition to natural antibodies, reducing tissue and organ damage caused by ICIs, by decreasing the titers of autoimmune antibodies in the circulation.^27^ Additionally, immunoglobulins may mitigate the “cytokine storm” during myocarditis.^28^

ICI‐induced myocarditis has been reported to be more common in males and in patients with diabetes mellitus, those undergoing combined targeted drug therapy, and malnutrition treated with anti‐PD‐1/anti‐PD‐L1 antibodies.^29^, ^30^ Studies have shown that most clinical symptoms of ICI‐induced myocarditis are atypical, with early onset, severe disease, and rapid progression^1^, ^31^; therefore, clinicians should be sufficiently aware of them. ICI‐related myocarditis can resemble many other cardiovascular diseases, such as acute coronary syndrome, which manifests as dyspnea, chest pain, or asthenia with elevated troponin levels, thus increasing the risk for diagnostic delay. However, the initial clinical examination may be misleading, and symptoms may mimic more common myocarditis that is not associated with ICIs. The cardiac biomarkers, cardiovascular imaging and electrocardiogram (ECG) are still essential in the diagnosis of ICI‐related cardiac myositis. The diagnosis of ICI‐induced myocarditis is suspected on the basis of the presence of new cardiac symptoms, new ECG changes (atrio‐ or intraventricular conduction defects, tachyarrhythmias, bradycardia), and/or a new increase in troponin levels. In particular, cardiac troponin T (cTNT) is sensitive for diagnosis and surveillance in myocarditis patients, and is associated with major adverse cardiomyotoxic events.^9^ Cardiovascular imaging, such as echocardiography, is essential to rule out other possible causes and confirm cardiac dysfunction.^32^ When there is a history of immunotherapy or clinical abnormalities, ICI‐related myocarditis should be considered after excluding acute congestive heart failure, acute coronary syndrome, and viral myocarditis. Once diagnosed, glucocorticoids should be administered according to disease severity and combined with plasma exchange, immunoglobulin(s), and other comprehensive treatment(s) to improve prognosis and reduce the fatality rate.

## 4 CONCLUSION

Results of this case series suggest that plasma exchange plus systemic glucocorticoids may be effective for the treatment of anti‐PD‐1/anti‐PD‐L1 antibody‐induced myocarditis in patients with cancer. Given the limited sample size in this study, however, additional larger‐scale, multicenter trials are required for further verification.

## AUTHOR CONTRIBUTIONS

*Design of the study*: Guibao Ke, Jie Xiao, Weijie Guan and Xiaomin Yu. *Data acquisition*: Peian Chen, Jing Luo, Yonghua Peng, Hongbo Peng, Yongzhang Huang, Junlin Huang, and Ningying Fu. *Data analysis*: Yuan Li, Bo Wang, and Yuqi Shang. *Draft of the manuscript*: Guibao Ke and Peian Chen. *Manuscript revision and final version approval*: Jie Xiao. All authors have read and approved the final manuscript.

## CONFLICT OF INTEREST STATEMENT

The authors declare no conflict of interest.

## Data Availability

All data generated or analyzed during this study are included in this article. Further inquiries can be directed to the corresponding author.
